# Variable Expression of Cre Recombinase Transgenes Precludes Reliable Prediction of Tissue-Specific Gene Disruption by Tail-Biopsy Genotyping

**DOI:** 10.1371/journal.pone.0001013

**Published:** 2007-10-10

**Authors:** Tim J. Schulz, Markus Glaubitz, Doreen Kuhlow, René Thierbach, Marc Birringer, Pablo Steinberg, Andreas F. H. Pfeiffer, Michael Ristow

**Affiliations:** 1 University of Jena, Institute of Nutrition, Jena, Germany; 2 German Institute of Human Nutrition, Potsdam-Rehbrücke, Germany; 3 University of Potsdam, Institute of Nutrition, Potsdam-Rehbrücke, Germany; The Babraham Institute, United Kingdom

## Abstract

The Cre/loxP-system has become the system of choice for the generation of conditional so-called knockout mouse strains, i.e. the tissue-specific disruption of expression of a certain target gene. We here report the loss of expression of Cre recombinase in a transgenic mouse strain with increasing number of generations. This eventually led to the complete abrogation of gene expression of the inserted Cre cDNA while still being detectable at the genomic level. Conversely, loss of Cre expression caused an incomplete or even complete lack of disruption for the protein under investigation. As Cre expression in the tissue of interest in most cases cannot be addressed *in vivo* during the course of a study, our findings implicate the possibility that individual tail-biopsy genotypes may not necessarily indicate the presence or absence of gene disruption. This indicates that sustained *post hoc* analyses in regards to efficacy of disruption for every single study group member may be required.

## Introduction

In recent years, the Cre/loxP-system has become the system of choice for the generation of conditional so-called knockout mouse strains, i.e. the tissue-specific disruption of expression of a certain target gene [1], [2]. As a body wide, i.e. non-conditional disruption of single genes frequently leads to premature lethality, conditional disruption is crucial to investigate the role of a protein by means of subdivided depletion in closely defined cell types.

Any deliberately chosen DNA sequence can be flanked with loxP-elements. LoxP-sites are inserted in the intronic spacers of exons which encode vital structures of the protein of interest. Cre mediated recombination subsequently leads to the deletion of the sequence between two loxP-elements and a truncated gene product if any at all.

Cre recombinase can be expressed in a given tissue or cell type under the control of a defined promoter fragment. Theoretically, expression occurs exclusively in the cell type, where the promoter usually is active [2]. By application of molecular biology methods, defined parts of the promoter can be linked to the cDNA of Cre recombinase which then is expressed in the same manner as the gene it is aimed to replace. Investigators working in this field, however, have found that Cre expression is often not strictly confined to the desired cells. Moreover, Cre transgenic mouse strains tend to display unspecific expression, and thus a knockout phenotype, in various cell types.

The mechanisms causing these undesired effects are widely unknown. In the first place, the activity of many promoters in most cases is not fully understood. Hence, there might be developmental stages or other environmental factors affecting the activity of a promoter that have not yet been characterized. When Cre expression occurs within an early embryonic stage, all cells derived from this lineage will carry the deleted gene.

Secondly, Cre transgenic mouse strains are mostly generated by random integration of a DNA construct comprising promoter and Cre cDNA into the host DNA. This may lead to unspecified genetic interactions, i.e. transactivations, at the site of integration which might be concurrently causative for unspecific Cre expression in many cell types.

Thirdly, a number of articles on the effects of integration site on the expression pattern of a given transgene have been published. Termed *position-effect variegation* (PEV), the phenomenon of mosaic expression has originally been described as the genetic cause of heterogeneously coloured eye-discs in mutants of *Drosophila melanogaster* [3]–[5].

Silencing is assumed to be due to site specific effects such as condensation of chromatin [6], [7], close proximity to the centromere [8], transgene orientation and methylation induced silencing [9]. Furthermore, expression levels of a given transgene appears to correlate inversely with ageing [10].

These previous publications altogether suggest that various processes may cause impaired expression of transgenes. We here demonstrated that this might, at least in some cases, precludes reliable prediction of tissue-specific gene disruption by tail-biopsy genotyping.

## Results

By means of site directed recombination, mouse models with a liver specific inactivation of various proteins employing Cre expression under the control of the Albumin promoter (*Alb*) have been generated [11]–[14]. Specifically for the *Alb* promoter driven expression of Cre recombinase, it has been reported, that the maximum level of recombination in hepatocytes occurs at two weeks of age [15]. We have used this line to generate a liver-specific knock-out of the frataxin gene [13], and have observed a unambiguously efficient rate of recombination in our initial study, i.e. all animals ever analyzed within the initial study group were *bona fide* knock-outs. Subsequently and to generate animals for several follow-up studies we observed inconsistencies between the phenotype initially observed [13] in comparison to animals that were derived from intercrosses of later generations carrying the *Alb* Cre construct.

**Figure 1:**
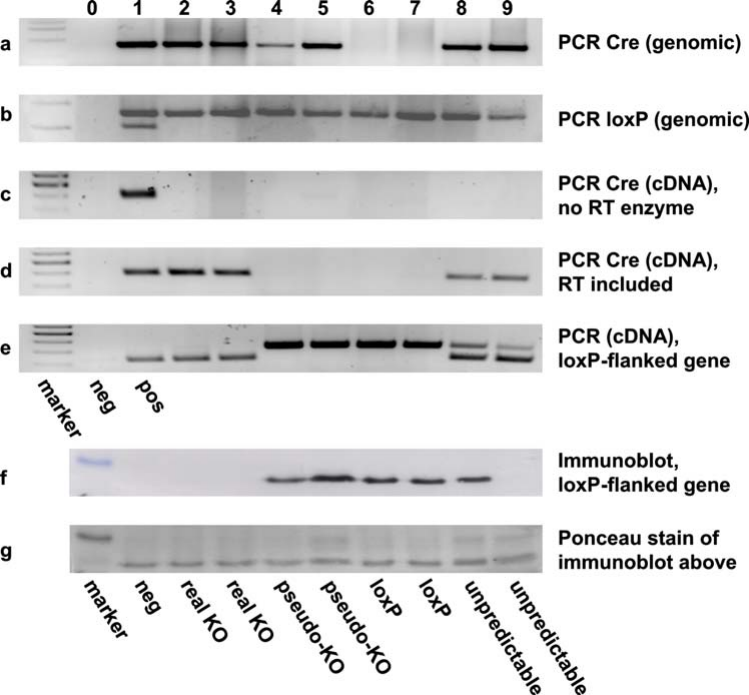
Genotyping and molecular phenotype analysis for hepatocyte specific gene inactivation in the mouse by the Cre/loxP-technique. (a) Cre-transgene detection by PCR with genomic DNA as template. Columns 0 and 1 depict negative and positive control, respectively (also applies to subsequent panels B to E). (b) Amplification of genomic DNA for detection of loxP flanked exon 4 of the frataxin gene. A double band depicts heterozygous genotype (column 1), one band at 545 bp only accordingly indicates homozygous loxP insertion (columns 2 to 9). (c) Control Cre-PCR from RNA without reverse transcription. (d) RT-PCR of Cre RNA expression. (e) RT-PCR directed at floxed exon 4 of the target gene. The shorter band stems from RNA with excised exon 4, the long band corresponds to the intact loxP flanked exon 4. (f) Immunodetection of Cre/loxP-targeted frataxin expression in the liver. (g) Ponceau staining of membrane before immunodetection to ensure equal loading of protein in all columns.

Therefore we started to systematically dissect these apparent inconsistencies. We first analyzed the presence of randomly integrated Cre transgene by PCR which gives rise to a single fragment of 387 bp based on previously described primers [16] derived from the original cDNA sequence of Cre recombinase (Fig. 1A, columns 1 to 5 and 8 to 9). The presence of a loxP-flanked exon 4 of the frataxin gene [17] was confirmed by PCR as previously described [16] and gives rise to either a double band for heterozygotes (Fig 1B, column 1) or a signal at 545 bp for animals homozygous for the loxP site (Fig. 1B, columns 2 to 9). In most laboratories, genotyping of Cre/loxP animals is achieved by PCR for Cre recombinase and the corresponding loxP-sites, and considered sufficiently informative while measurement of RNA expression and/or immunodetection methods for the presence of disruption is usually performed for a limited number of animals only. Accordingly, panels A and B of Fig. 1 depict the typical genotyping result, and would lead to the assumption that column 1 reflects a hemizygous tissue-specific disruption, while columns 2 to 5 and 8 to 9 would indicate the presence of disruption of both alleles, i.e. a knock-out.

To further evaluate the actual expression of the Cre transgene, we performed RNA expression analysis. After DNAse digestion and subsequently performed reverse transcription, cDNA was subjected to PCR employing the same primers as for detection of genomic Cre cDNA [16]. While omission of reverse transcription (Fig. 1C, columns 2 to 9) gave no specific PCR signal indicating that genomic DNA was absent, RT-PCR of all samples (Fig. 1D) gave specific signals indicating the presence of Cre recombinase mRNA in the originating sample for a subset of samples (Fig. 1D, columns 2, 3, 8, 9) only. This indicates that despite the presence of a genomic Cre signal (Fig. 1A) some liver specimen did not express Cre recombinase (Fig. 1D, columns 4 and 5). The imbalance presented by the misleading genomic Cre signal could be confirmed by RT-PCR against the targeted exon 4 of the gene under investigation. Only in those animals, where Cre RNA could be detected, excision of the fourth exon took place in liver specimen (Fig. 1E, columns 2, 3 and partially at 8 and 9). However, in some animals, excision was incomplete and lead to a partial retention of the intact target gene (Fig. 1E, columns 8 and 9). This finding might correlate with the level of Cre expression, as in these latter animals, signals derived from Cre RT-PCR were found to be weak (Fig. 1D, columns 8 and 9).

To further extend our findings in regards to the lack of expression of Cre in certain animals, we performed Western blot analyses to assess hepatic expression levels of the targeted protein, frataxin. In those animals which showed Cre transcription and complete target gene truncation, the knockout was detectable and complete on a translational level (Fig. 1F, columns 2 and 3). However, concurrent with the lack of Cre mRNA, we found a normal expression of frataxin protein in those animals that where genotyped as knockouts, but had no Cre expression or truncation of the target gene-RNA (Fig. 1F, columns 4 and 5). More surprisingly, we found a complete lack for the expression of the targeted protein in one of the animals with incompletely truncated RNA (Fig. 1F column 9), while the other apparently had no reduction in protein expression at all (Fig. 1F, column 8) despite the fact that no gross differences in regards to Cre (Fig. 1D) and frataxin (Fig. 1E) transcripts were detectable.

## Discussion

Our current findings suggest the possible involvement of several distinct mechanisms, which lead to the observed silencing of transgene expression. Since the Cre cDNA has been randomly integrated into the genomic DNA [11], it is likely that not all copies integrated into the genome have the same transcriptional activity. This might be due to various silencing effects of the neighbouring DNA-sequences. One possible model to explain the apparently contradictory findings would include the event of homologous recombination of the transgene flanking regions, in which inactive copies of the transgene segregate from those still active. This process could be a result of multiple recombination events throughout a number of consecutive generations, i.e. breeding. As a consequence, the inactive copy of the transgene could still be detected by genomic PCR while no expression of the transgene would take place.

Recently, in the model organism *Drosophila melanogaster*, it has been proposed, that the Argonaute proteins AGO1 und AGO2 play a central role in post-transcriptional silencing [18]. Taking into account the conflicting phenotypes in some animals with only partial recombination of the target gene (Fig.1, panels E and F, columns 8 and 9), our data tentatively suggest, that transcript degradation could at least play a possible role in silencing expression of Cre recombinase.

From retrovirus-mediated transgene expression it is known that methylation of the long terminal repeat region takes place. Furthermore, binding of transcriptional inhibitors to responsive elements in the transgenic construct results in reduced expression of the transgene [19]–[21]. This tentatively indicates the possibility of silencing mechanisms acting in parallel on genetic and transcriptional levels.

Taken together, the data presented here indicate that, although misleadingly being detectable at a genomic level, expression of the Cre transgene may be completely abolished in a subset of mice. Further experiments will be required to elucidate the mechanism(s) responsible for these effects. These findings highlight the importance of sustained and comprehensive monitoring of the presence of disruption on a transcriptional or translational level for tissue-specific knock-out models.

## Materials and Methods

### Maintenance and genotyping of animals

Animals were generated [17] and maintained [16] as previously described.

### Nucleic acid isolation and amplification

Isolation of genomic DNA and RNA was performed as previously described [16]. Amplification of genomic DNA and reversely transcribed RNA was performed as described [16] except that newly isolated RNA was digested with DNAse I (Roche, Basel, Switzerland) and the restriction enzyme AluI (Roche), an enzyme additionally used to ensure complete removal of contaminating genomic DNA. Subsequently, samples were loaded onto a column to separate RNA from enzyme and digestion buffer (RNeasy Cleanup Kit, Qiagen, Weiden, Germany).

### Protein Extraction and Immunodetection

Protein samples were prepared as described [22]. For SDS-PAGE, 50 µg of denatured protein-lysate was loaded on each lane of a 16% gel. Separation of proteins and frataxin expression analysis were performed as described previously [22] with a polyclonal antibody directed against mouse frataxin [17].
